# Editorial: Vaccines on mucosal immunity

**DOI:** 10.3389/fmicb.2025.1647592

**Published:** 2025-07-29

**Authors:** Alexander Suvorov, Julio Villena, Kathleen Hefferon

**Affiliations:** ^1^Department of Molecular Microbiology, Institute of Experimental Medicine, Saint-Petersburg, Russia; ^2^CERELA, San Miguel de Tucumán, Argentina; ^3^Department of Microbiology, Cornell University, Ithaca, NY, United States

**Keywords:** immunology, mucosal immunity, infectious disease, vaccines, microbiology

Vaccines are critical to our successful management of infectious diseases. Many pathogens enter the body via mucosal surfaces, yet many vaccines are delivered parenterally or by other means and do not elicit mucosal immunity as a first line of defense. As such, attention has been placed on the further development of mucosal vaccines. Mucosal vaccines can be cheaper and easier to administer. Despite these advantages, however, the number of approved orally delivered mucosal vaccines is limited, with a chief challenge being stability through the harsh environment of the gastrointestinal tract. In this Research Topic, advancements in vaccines that target mucosal immunity are explored through the manuscripts of several authors and are summarized below.

An article by Buzás, titled “Bacterial carbonic anhydrase as a candidate vaccine target against *Helicobacter pylori*,” describes a novel vaccine strategy to protect individuals from bacteria linked to stomach ulcers and gastric cancer. In this study, the author proposed identifying bacterial *H. pylori* carbonic anhydrase CA (*Hp*CA) epitopes and generating targeted antibodies. Once these epitopes have been developed, the author proposes using mRNA vaccine technology to target the HpCAs since they are critical to the bacteria's survival. Such a vaccine could be a game changer for protection against *H. pylori* infection.

Author De et al. presents “A novel oral vaccine delivery system for enhancing stability and immune protection: bacterium-like particle with functional coating.” Bacterium-like particles (BLPs), generated by heat-acid treatment of lactic acid bacteria and displaying antigens on the cell surface, have great potential as immune enhancers and antigen delivery systems. Professor De's paper describes a simple self-assembly mechanism for lipid membranes onto the BLP surface that enables the vaccine, when delivered orally, to survive the harsh environment of the gastrointestinal tract. This novel approach and the positive outcomes of initial trials could enable further use of BLPs as vaccines that target the mucosal immune system.

Austriaco submitted the mini review “Yeast oral vaccines against infectious diseases,” which offers a fresh perspective on the opportunities and challenges for yeast-based oral vaccine delivery. Included are the advantages of recombinant yeast in the form of whole cells that are orally consumed to deliver vaccine antigens to the gut. This review begins with a discussion of the challenges associated with oral administration of vaccines and the distinct benefits offered by whole yeast delivery systems over other delivery systems. The mini review then provides examples of various yeast oral vaccines and shows proof of concept of their promise.

A paper by Medeiros et al., titled “Oral polio revaccination is associated with changes in gut and upper respiratory microbiomes of infants,” explores the fate of infants' microbiota who may no longer receive the live-attenuated oral polio vaccine (OPV) after the virus is eradicated from the planet. This study involved deep sequencing of 16S rRNA of both fecal and nasopharyngeal microbiomes in revaccinated Bissau-Guinean infants and comparing these results to those of infants who were not revaccinated with OPV. The study demonstrated a greater increase in microbial diversity for infants who had been revaccinated compared to those who had not. Revaccinated infants also showed a reduction in potentially pathogenic/opportunistic bacteria such as *E. coli* or *Shigella* within the gut and *Streptococcus/Hemophilus* in the respiratory microbiota compared to controls. The authors concluded that, in general, revaccination with OPV was associated with a healthier microbiome composition along with a decrease in pathogen presence.

Each of these articles offers a novel approach to the design and efficacy of vaccines which could provide a robust mucosal immune response. Each of these approaches has potential and could be used to target several pathogens. Hopefully, these approaches will gain traction and provide solutions to the pathogens we face today, from *H. pylori* in the stomach to *Streptococcus* in the respiratory tract and many others.

